# Tuberculosis sanatorium treatment at the advent of the chemotherapy era

**DOI:** 10.1186/s12879-020-05539-w

**Published:** 2020-11-11

**Authors:** Erin D. Zwick, Caitlin S. Pepperell

**Affiliations:** 1 Department of Population Health Sciences, UW-Madison, Madison, USA; 2 Departments of Medicine and of Medical Microbiology and Immunology, School of Medicine and Public Health, UW-Madison, Madison, USA

**Keywords:** Tuberculosis, Sanatorium, Length of treatment, Tuberculosis treatment

## Abstract

**Background:**

The discovery of antibiotics in the mid-twentieth century marked a major transition in tuberculosis (TB) treatment and control. There are few studies describing the duration of TB disease and its treatment from the pre-chemotherapy era and little data on how these treatments changed in response to the development of effective antibiotics. The goal of this research is to understand how inpatient treatment for high incidence populations, the First Nations peoples of Saskatchewan, Canada, changed in response to increasing availability of antibiotics effective against TB. We expected that as treatment regimens transitioned from convalescence-only to triple antibiotic therapy, the length of inpatient treatment would shorten.

**Methods:**

Analyses were performed on records of sanatoria admissions and discharges occurring between 1933 and 1959 in Saskatchewan, Canada. Year of antibiotic discovery was taken as a proxy for treatment regimen: no chemotherapy (pre-1944), mono-therapy (Streptomycin, 1944–1946), dual-therapy (Streptomycin and PAS, 1946–1952), and triple-therapy (Streptomycin, PAS, and INH 1952-). A pooled linear regression of log-transformed length of first admission as predicted by year of admission was modeled to assess the relationship between admission length and year of admission, corrected for clinical and demographic variables.

**Results:**

First admission length increased 19% in the triple-therapy era as compared to the pre-chemotherapy era, from 316 days (10.4 months) to 377 days (12.4 months). After the discovery of INH (1952), we find statistically significant increases in the proportion of successfully completed therapies (0.55 versus 0.60, *p* = 0.035), but also in patients who left hospital against medical advice (0.19 versus 0.29, *p* < 0.0001), indicating that as hospitalizations lengthened, more patients chose to discharge without the sanction of their physician. The readmission rate increased from 10 to 50% of all admissions while the province-level TB-specific death rate fell from 63.1 per 10,000 in 1933 to 4.7 per 10,000 in 1958.

**Conclusion:**

Counterintuitively, we find that the length of first admissions increased with the discovery of TB-treating antibiotics. Increasing admission volume and readmission rate indicate an intensification of inpatient TB treatment during this era. These analyses provide a novel estimate of the effect of changing treatment policy on sanatorium admissions in this population.

**Supplementary Information:**

The online version contains supplementary material available at 10.1186/s12879-020-05539-w.

## Background

In the early to mid-twentieth century, First Nations communities of Western Canada experienced severe epidemics of tuberculosis (TB) [1–3] . TB-specific death rates in First Nations communities were as much as ten to twenty times higher than the rates in non-First Nations populations during this time [4]. The discovery of TB chemotherapy, along with intensified efforts at prevention and care, resulted in dramatic reductions in TB mortality by the 1960s [1].

Streptomycin, the first antibiotic used to treat TB, was discovered in 1944 [1, 2], followed by para-aminosalicylic acid (PAS) in 1946 [1, 2] and isoniazid (isonicotinic acid hydrazide, or INH) in 1952 [1, 2]. By the 1960s, at least a dozen antibiotics with activity against TB had been discovered, resulting in more effective and quicker treatment [2]. Prior to streptomycin, TB treatment options were limited to convalescence at a TB sanatorium and a suite of surgical techniques [1]. Hospitalization typically lasted until self-cure or death, which could take years [5]. Early drug treatments were not a panacea; for example, streptomycin has toxic side effects and monotherapy is ineffective over the long term as antibiotic resistance readily emerges in TB infections [2]. The addition of PAS in the dual-therapy era and INH in the triple-therapy era increased the effectiveness of chemotherapy and the combination of streptomycin, PAS, and INH served as the primary TB treatment regimen for almost two decades [2].

Antibiotic treatments for TB rendered convalescent sanatorium treatment obsolete [1]. However, even as TB treatment for other populations shifted to the outpatient setting, First Nations patients continued to be institutionalized for treatment [1, 2]. After World War II, more resources were allocated for medical services to First Nations populations, including beds in TB sanatoria [1]: in 1937, there were approximately 100 First Nations patients being treated for TB in hospitals and sanatoria in Western Canada, whereas by 1953 there were 2975 [1]. By the mid-1960s, outpatient TB care was made available to First Nations populations, at least a decade after becoming available to the rest of the population [1].

Using medical records from three sanatoria in Saskatchewan, Canada between 1933 and 1959, we investigated the effects of newly available drugs on hospitalizations of First Nations’ individuals with TB. In particular, we measured the length of sanatorium admission as a function of time, a proxy for the changing treatment methods. We also examined discharge classifications over this interval to ascertain changes in likelihood of therapy completion. Finally, we looked at readmission rates and mortality rates to determine survivorship during different treatment eras.

## Methods

### Dataset

We analyzed admission and discharge records from three tuberculosis sanatoria in Saskatchewan from the archives of the Saskatchewan Lung Association. The dataset consists of records of 1966 discharges that occurred between 1935 and 1959, and 2040 admissions that occurred from 1933 to 1959. In the original dataset, discharges were described as resulting from a patient completing therapy, dying, being ‘sent home to die’ or lost to follow up. We further classified the discharges post-hoc as resulting from a first admission or a readmission. Classification as a discharge arising from readmission was made if the discharge record contained data on previous admissions. Discharges with no such data were cross-referenced with admission records and, based on evidence of previous admissions, were classified as either a discharge arising from first admission or a discharge arising from readmission. Hence, discharges from first admission can be taken to be discharges of patients without a prior record of admission or discharge. Admission date was determined by subtracting admission length from discharge date, then cross-referencing the resulting date with original admission records for verification if possible.

A subset of 854 records from first discharges classified as therapy complete were used for more rigorous statistical analysis of patterns in admission length over time (Table 1). This subset was limited to first admissions to capture only incident cases and limited to completed therapies to remove observations that were censored by early death or discharge before therapy completion, thus limiting the model to the effect of treatment regimen on length of treatment. Losses to follow up from early discharge were described in the original historical data as ‘disciplinary’, which we interpret as eviction from the sanatorium, and ‘AWOL’ or ‘run away’, which we interpret as leaving against medical advice. These early discharges were analyzed separately.

**Table 1:** Descriptive statistics for categorical variables. (*n* = 854)

Variable	Frequency (percent)
Institution	
1	183 (21.4)
2	356 (41.7)
3	59 (6.9)
Missing	256 (30.0)
Sex	
Female	465 (54.4)
Male	387 (45.3)
Missing	2 (0.3)
Diagnosis	
Pulmonary	606 (71.0)
Extra-pulmonary	235 (27.5)
Disseminated/miliary	13 (1.5)
Smear status on admission	
Positive	188 (22.0)
Negative	525 (61.5)
Missing	141 (16.5)
Smear status on discharge	
Positive	15 (1.8)
Negative	839 (98.2)
Marital status	
Married	215 (25.2)
Single	558 (65.3)
Other	12 (1.4)
Missing	69 (8.1)

### Statistical analysis

We tabulated descriptive statistics for the primary dataset of interest (discharges from completed therapies). All analyses were performed in R (version 3.2.5). Data missing from the subset of discharges following completion of therapy were imputed using the mi package (version 1.0) in R according to methods outlined in Su et al. [6]. Pooling over all imputed datasets, we fit a generalized linear model to the log-transformed outcome of length of admission as predicted by year of admission, smoothed with natural cubic splines. The model was adjusted for age, sex, institution of admission, diagnosis, and smear status on admission and discharge. The model included an interaction term for age and sex. Due to the log-transformation of the outcome, results are reported as median length of stay, taken to be approximately the geometric mean produced by the model. The 2040 admission and readmission records were used to calculate the rate of readmission over the data collection period. TB-specific death totals were recorded from the Bureau of Epidemiology registry files from the Library and Archives Canada [7] and death rates were then calculated using census data.

## Results

**Fig. 1:**
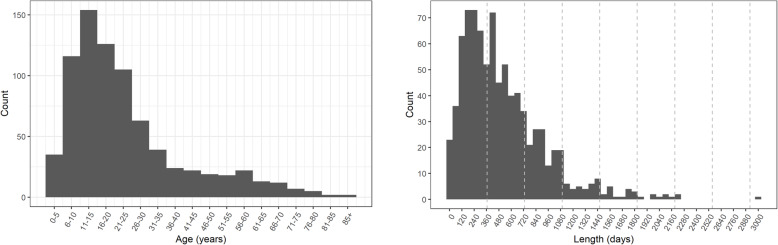
(L) Age distribution (*n* = 784, 70 (8.2%) observations missing) in 2-year bins. (R) Admission length in days in 60-day bins. Year cutoffs denoted by dashed lines

Table 1 summarizes the categorical variables describing discharges arising from first admissions and ending in completed therapies; distributions of continuous variables are shown in Fig. 1. Institution was the variable with the highest proportion of missing entries, at 30% of observations missing this descriptor. Diagnosis, smear status on discharge, and length of admission were all complete. Marital status was used for data imputation but was not used in statistical analyses as it is not a suspected confounder of the relationship between admission length and year of admission. Continuous variables, age and admission length, are shown in Fig. 1: distributions of both are right skewed. Overall, 48% of records in the dataset have one or more descriptors missing.

The data appear to be internally coherent, though there are 15 cases with apparently discordant data in that they are recorded as therapy complete but were smear positive on discharge. We do not know if these records are erroneous and if they are, which categorization is in error (i.e. smear status or discharge classification) so we retained them in our analyses.

**Fig. 2:**
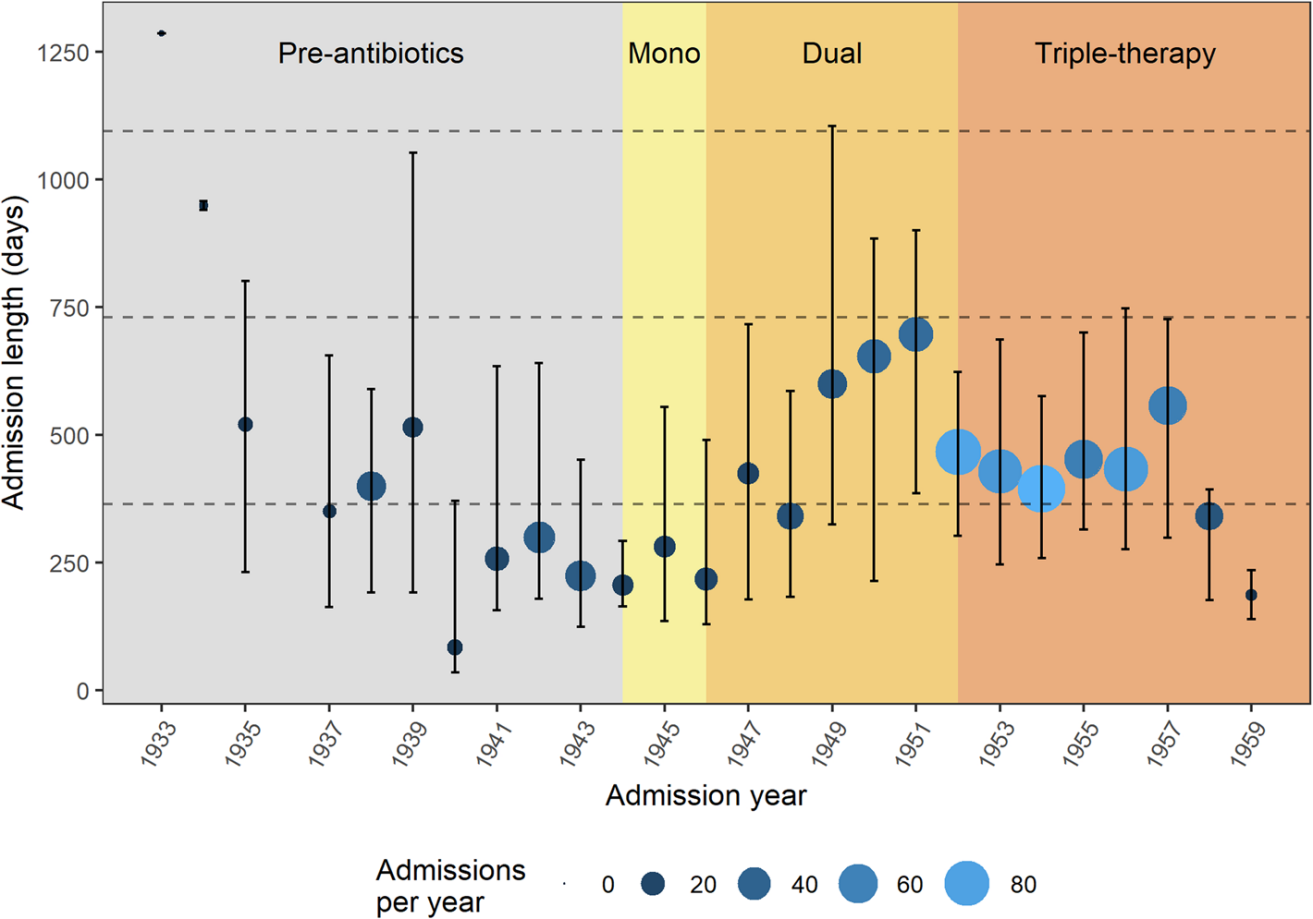
Median admission length (circles) with inter-quartile range (bars) over time. Circle size reflects total annual first admissions and horizontal lines represent year cutoffs. No first admissions were recorded in 1936

Length of first admission appears bimodal with peaks in the early 1940s and early 1950s (Fig. 2). The late 1940s and early 1950s saw an increase in both total first admissions and length of first admission. The total number of first admissions per year peaked at 89 in 1954, while median first admission length peaked in 1951 at 697 days, considering years with at least five first admissions recorded (Fig. 2). Admissions from 1958 and 1959 appear shorter due to censoring--discharge data were not available after 1959.

**Fig. 3:**
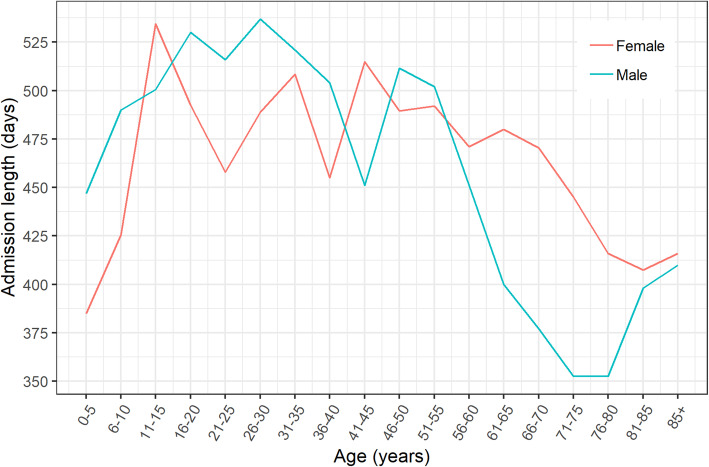
Median admission length by sex and age on admission

Length of first admission varied across confounding variables, including institution of admission, admission diagnosis, and admission smear status. Length of admission did not vary significantly between the sexes, though our results suggested a possible interaction between age and sex as a predictor of first admission length: median length of first admission appears to drop off more steeply for older males in the dataset than for older females (Fig. 3).

We performed a pooled linear regression after multiple imputation. We qualitatively analyzed the data for patterns in missingness prior to imputation, with particular emphasis on missingness patterns as related to the outcome variable, length of admission. We did not detect obvious patterns of missingness, except that institution was not regularly recorded in early years (Figure S1). From published analyses of related historical data, we find that all institutions in our dataset were open and accepting First Nations patients for the duration of the study period [1, 2]. Therefore, we assume the data are missing at random and suitable for multiple imputation.

The mi package [6] in R (version 3.2.5) was used to impute 48 datasets, one for each percent missing data, using settings recommended in Su et al. The imputations were performed across four chains then re-imputed four times until $\hat{R}$, a summary statistic measuring convergence between imputed and original data, converged to approximately 1.00 for all means and standard deviations (Additional File 1 Table S1).

**Fig. 4:**
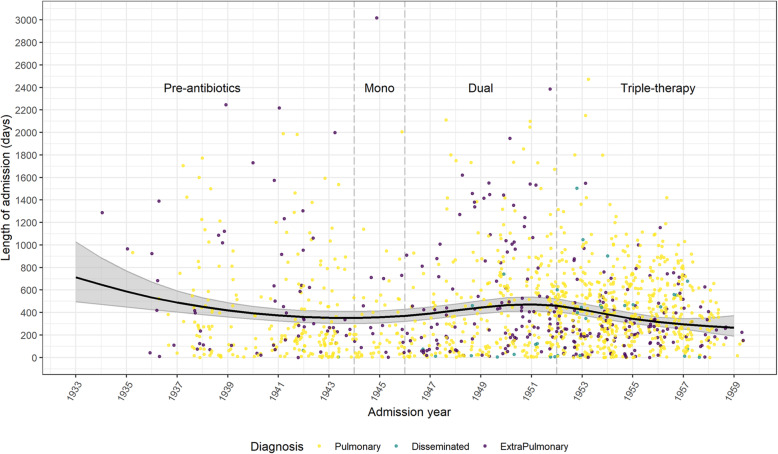
Results from one imputed dataset showing length of admission plotted over time with mean admission length over time as predicted by admission year smoothed with natural cubic splines

Results from univariate analysis of one of the imputed datasets are shown in Fig. 4. We find that average admission length trended downward for admissions through 1944 before increasing through 1952 then stabilizing in the later 1950s. This same pattern is evident in the pooled results, corrected for other confounders (Table 2, Additional File 1 Table S2).

**Table 2:** Median length of admission as predicted by model for one imputed dataset

	Days admitted as predicted by model	95% CI
Era (Year)*		
Pre-chemotherapy (1933–1944)	316	(258, 387)
Mono-therapy (1944–1946)	284	(233, 345)
Dual-therapy (1946–1952)	392	(328, 469)
Triple-therapy (1952-)	377	(323, 439)
**Diagnosis**		
Pulmonary	392	(328, 469)
Extra-pulmonary	397	(334, 472)
Disseminated	520	(310, 870)
Institution*		
1	291	(244, 346)
2	392	(328, 469)
3	329	(269, 402)
Smear status on admission**		
Positive	615	(506, 745)
Negative	392	(328, 469)
**Smear status on discharge**		
Positive	248	(203, 301)
Negative	392	(368, 476)
**Sex**		
Female	392	(328, 469)
Male	418	(368, 476)

Note: all estimates are from one imputed dataset using the model specified for the pooled analyses. Unless otherwise stated, estimates are for a female patient in 1949 with pulmonary TB at Institution 2, smear negative on admission and discharge. Years representative of era were taken to be the midpoint between era designations

* *p* < 0.005 in pooled analysis

** *p* < 0.0001 in pooled analysis

We regressed the log-transformed length of admission on year of admission smoothed by natural cubic splines adjusted for smear status on admission and discharge, diagnosis on admission, age, sex, the interaction between age and sex, and institution for each of the datasets, then pooled results from all 48 models using methods in the mi package. We found that in addition to the intercept, year of admission (*p* = 0.003), institution of admission (*p* = 0.002), and admission smear status (*p* < 0.0001) were significant predictors of admission length in the pooled model (Additional File 1 Table S2). Predicted admission length is lowest in the mono-therapy era and highest in the dual-therapy era (Table 2), with a 19% increase in admission length in the triple-therapy era compared to the pre-chemotherapy era. Patients at Institution 2 were admitted 35% longer than those at Institution 1 and 19% longer than those at Institution 3. The interaction between age and sex was also significant (*p* = 0.02), with older, male patients predicted to have shorter admissions.

Smear status on admission was highly significant in the model. From the model, patients sputum smear positive on admission were predicted to have been admitted approximately 57% longer than smear negative patients. Though not significant in the model (*p* = 0.52), we find that patients with disseminated diagnosis were admitted 30% longer than patients diagnosed with pulmonary or localized extra-pulmonary disease. Both disseminated disease and smear positive diagnoses are considered more severe [8], so we expect that a robust model will reflect longer admission lengths in these two categories. Smear status on discharge was not significant in the pooled analysis (*p* = 0.31).

**Fig. 5:**
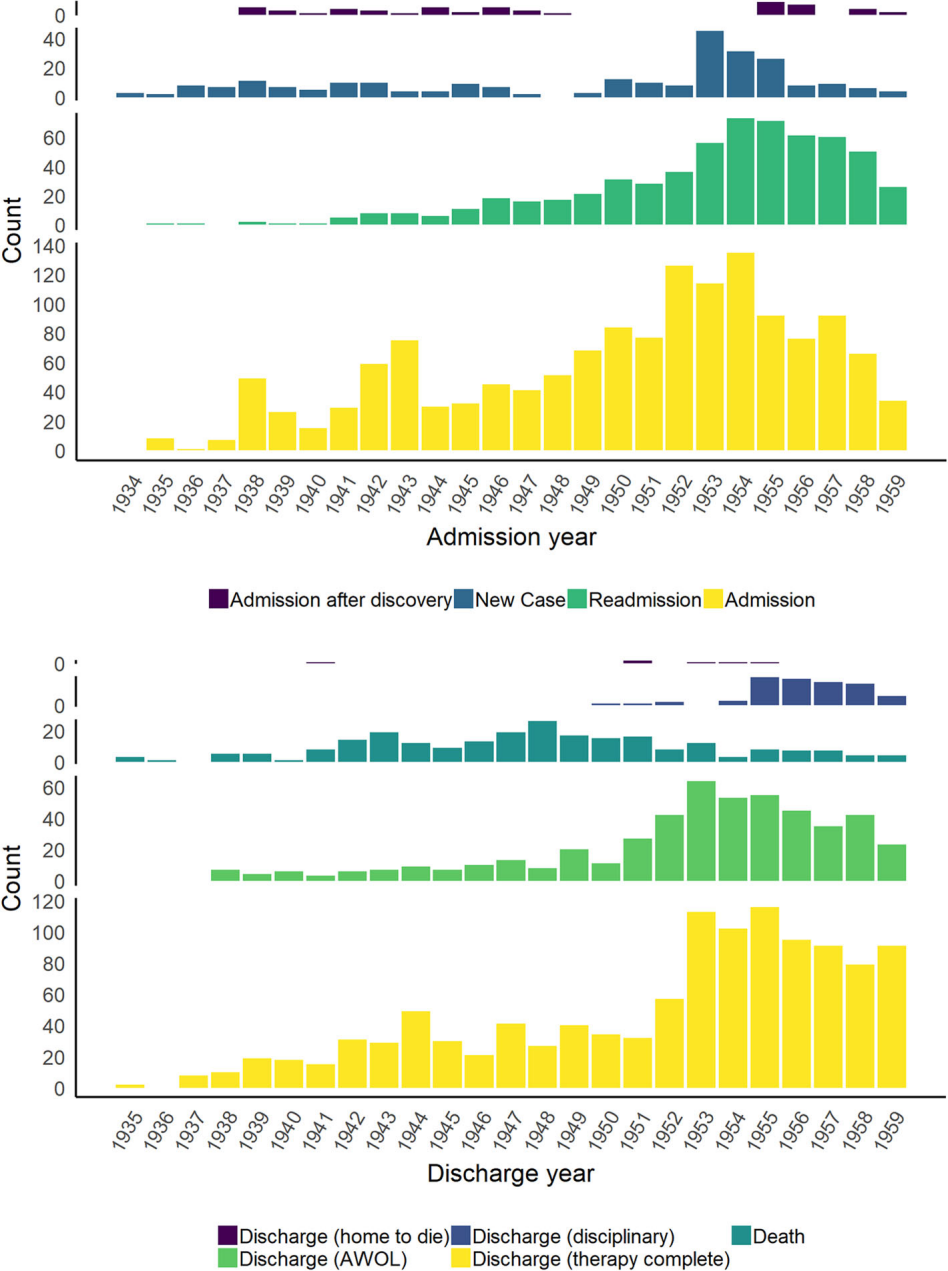
Distribution of admissions (*n* = 2040) (top) and discharges (*n* = 1966) (bottom) by type over study period

Analysis of the un-imputed full dataset of admissions (*n* = 2040) and discharges (*n* = 1966) is shown in Fig. 5. Cases were classified in the original dataset as admission, readmission, new case, and admission after discovery. ‘New cases’ were new diagnoses detected in the population but not admitted to a sanatorium (i.e. observed as outpatients) while ‘admissions after discovery’ were ‘new cases’ identified in the past, but not admitted at time of diagnosis, now being admitted to a sanatorium. We find that admissions peaked in 1954 while discharges, including early discharges and deaths, peaked the following year. Losses to follow up from early discharge included discharges due to eviction (described as ‘disciplinary’ in the historical data) or leaving against medical advice (described in the historical dataset as ‘running away’ or ‘AWOL’) increased during the late 1940s and early 1950s (Fig. 5). We find a statistically significant increase in the proportion of discharges classified as therapy complete after 1952, which defines the onset of the triple-therapy era. We also find significant increases in the proportion of discharges against medical advice and due to eviction after 1952. Additionally, we find a highly significant decrease in the proportion of discharges due to death after 1952 (Table 3).

**Table 3:** Two-tailed Z-test results for difference in proportion of discharge classification before and during triple-therapy era

Discharge classification	Proportion before 1952 (*n* = 732)	Proportion after and including 1952 (*n* = 1234)	Difference in proportion two tailed Z-test (*p* value)
Therapy complete	0.55	0.60	0.035
Death	0.25	0.04	0.00
Lost to follow up (‘left against medical advice’)	0.19	0.29	*p* < 0.0001
Lost to follow up (‘eviction’)	0.003	0.06	*p* < 0.0001
Home to die	0.004	0.002	0.41

**Fig. 6:**
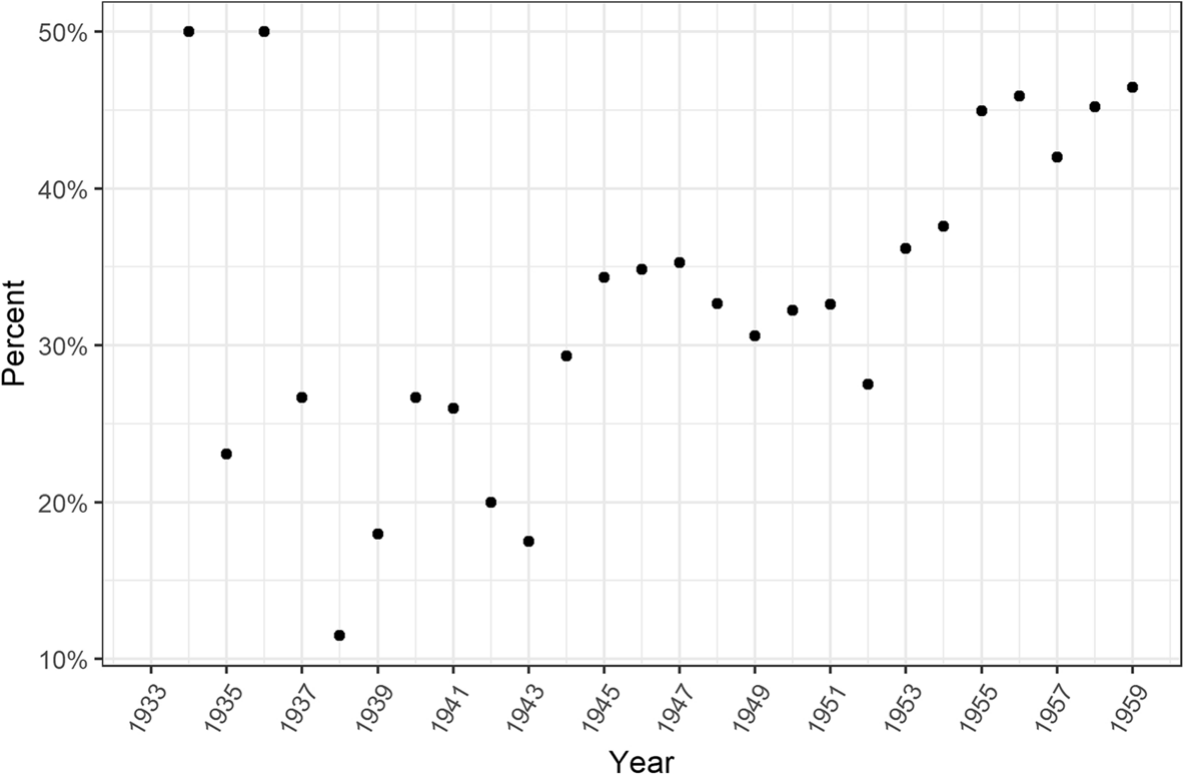
Proportion of readmissions increase as new treatments are implemented

**Fig. 7:**
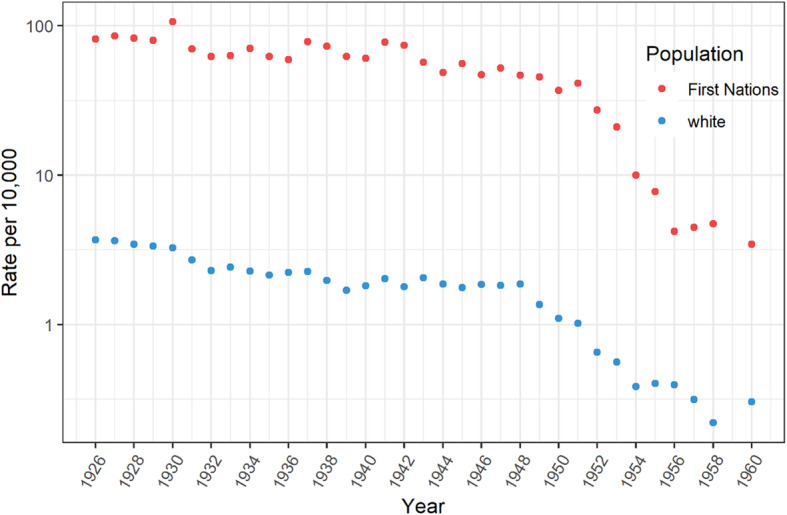
Province-level tuberculosis-specific death rate for First Nations and total population of Saskatchewan from 1926 to 1960

Over the observation period, readmissions accounted for an increasing proportion of total admissions, accounting for almost 50% of all admissions in the late 1950s versus approximately 25% of admissions in the late 1930s (Fig. 6). Simultaneously, the tuberculosis-specific death rate decreased in both the First Nations and total population of Saskatchewan. Though the trends follow the same general pattern, the death rates for the two populations are orders of magnitude different, with a maximum death rate of 4.8 per 10,000 in the total population in 1926 and a maximum death rate of 106.0 per 10,000 in the First Nations population in 1930 [7] (Fig. 7). The death rates in both populations declined gradually in the 1930s through the early 1940s before a steep decline that began in the late 1940s, coincident with the availability of antibiotics to treat TB. TB-specific death rates for both populations were not adjusted for age, which was not reported in the historical data.

## Discussion

The discovery of effective tuberculosis (TB) drugs, starting with streptomycin in 1944, resulted in dramatic declines in deaths due to the disease. In the First Nations population of Saskatchewan, the TB death rate fell from 63.1 per 10,000 people in 1933 to 21.0 per 10,000 in 1953 [7] (Fig. 7). Untreated TB often has a protracted course, with estimates of the average time to death or self-cure in the range of 2 to 3 years [5]. Our a priori expectation was that the length of sanatorium admission would decrease after the discovery of effective TB drugs. Instead, we found evidence suggesting that inpatient treatment of First Nations TB patients expanded after novel drug treatments became available.

First Nations TB patients in the mid twentieth century endured prolonged hospitalizations: median 396 (range: 3–3017) days for admissions occurring between 1933 and 1959. Over the course of the study interval the number of first admissions increased, and the length of first admissions generally increased and then stabilized in the late 1950s. We also found that readmission rates increased during the data collection period, from approximately 35% of all admissions in the mono- and dual-therapy eras to over 50% in the triple-therapy era. Admission length fluctuated across eras associated with progressively more effective TB treatments: a demographically similar patient would expect to be admitted for 32 (10.2%) fewer days in the mono-therapy (streptomycin) era compared to the pre-chemotherapy era, suggesting that use of the drug was associated with a shortened interval to clinical cure and/or death. Reversing that trend, patients in the dual-therapy (streptomycin and PAS) and triple-therapy (streptomycin, PAS, and INH) era were admitted on average 76 (24%) and 61 (19%) days longer than in the pre-chemotherapy era respectively. This could represent evolving goals of treatment and/or changes in the standard for what constituted clinical cure. The lower death rate and rising readmission rate in the post-chemotherapy era indicate that advances in TB treatment helped keep patients alive, even if, as is the case with monotherapy [9], the treatment may not have been effective enough to cure the patient. We note that some patients admitted in the mono-therapy era, which as defined is only 2 years, likely changed regimens during their admission given that median admission lengths during that era approached 1 year (Fig. 2). However, considering all eras prior to effective therapy, we find significant change in admission before and after the advent of triple-therapy: after 1952, and the discovery of INH, we find a significant increase in the proportion of patients who completed therapy at these three sanatoria, indicative of the effectiveness of triple-therapy (Table 3).

We also found that admission length varied as a function of smear status on admission, institution of admission, and in response to the interaction between age and sex. This variation is expected given what we know of TB clinically (longer treatment is required for smear positive pulmonary disease [8]) and from the underlying data (Fig. 3, Table 1). The above predictor variables collectively accounted for as much variability in admission length as year of admission. The most striking difference in admission length is across smear status, with patients smear positive on admission staying on average 57% longer than demographically similar smear negative patients. Smear positivity is an indicator of disease severity [8] and this result indicates that our model performs as expected. Though not statistically significant in the model, we find that treatment length varied across diagnoses, with the longest predicted treatment length (520 days) associated with the most severe diagnosis, disseminated disease.

The post-World War II period in Western Canada was a period of intensification of TB prevention and care efforts that included active surveillance of the entire population, suggesting that delays between disease onset and sanatorium admission may have been relatively brief during this interval [1] and total disease duration may not have been markedly longer than the treatment duration estimated here. Frequently cited estimates obtained for modern, post-chemotherapy data, of the duration of untreated, active TB infection before self-cure or death are approximately 2 years [5]. However, a meta-analysis of pre-chemotherapy studies of tuberculosis incidence, prevalence, and mortality found that the average duration of untreated TB in HIV-negative patients was approximately 3 years until death or self-cure [5]. From the unmodeled data, we find median admission length was 297 days (9.8 months) in the pre-chemotherapy era, 212 days (7.0 months) in the mono-therapy era, 518 days (17.0 months) in the dual-therapy era, and 437 days (14.4 months) in the triple-therapy era. Model-adjusted median admission lengths from the regression analysis are: 316 days (10.4 months) in the pre-chemotherapy era, 284 days (9.3 months) in the mono-therapy era, 392 days (12.9 months) in the dual-therapy era, and 377 days (12.4 months) in the triple-therapy era. This suggests that in the nascent antibiotic era, the effect of TB treatment on shortening disease duration was modest. It is striking that greater than 1 year of hospitalization was deemed necessary to achieve clinical cure, even in the triple therapy era. By comparison, current clinical practice guidelines recommend 26 weeks (6.5 months) of combination antibiotic therapy for the treatment of drug susceptible pulmonary TB [10].

The middle decades of the twentieth century were a time of transition in TB treatment. In 1948, the World Health Organization (WHO) began a domiciliary drug program that recommended TB treatment take place in an outpatient setting [2]. However, fully outpatient treatment was not achieved until the next decade: treatment guidelines from the 1950s, including recommendations from a clinical study of TB treatment in 1953 [11], recommended triple-therapy antibiotic courses of 18–24 months to achieve cured status [2]. It was recommended that treatment be administered in an inpatient setting at least until the patient was no longer infectious [2]. By 1959, prominent TB researchers were confident TB could be treated from start to end on an outpatient basis [12] and by 1965, TB treatment in Canada transitioned from inpatient treatment to a domiciliary drug program [2]. We find that the First Nations patients were treated as inpatients during this transition decade for what appears to be the entire recommended course of treatment (average 16.3 months). Sanatorium treatment declined rapidly in Canada starting in the mid-1950s until most sanatoria were closed in the late 1960s [1, 2].

We found that admission lengths increased as treatment methods became more effective [11, 13, 14] in concert with an expansion in the total number of First Nations patients admitted to sanatoria. Medical historians have pointed to striking differences in TB control policy applied to First Nations populations in this era [1, 2, 4]. One possible explanation of our observation of intensified inpatient treatment of First Nations patients with TB at the advent of the chemotherapy era is a shift to outpatient TB treatment for the non-First Nations population [2], made possible by the discovery of PAS in 1946 and INH in 1952 [1], which were taken orally, as opposed to streptomycin, which was taken intravenously [2]. As non-First Nations patients were shifted to outpatient treatment and the sanatorium system risked fading to obsolescence, a proposal to open 1390 newly vacated beds to First Nations patients was put forth in 1945 [1]. Prior to the 1940s, sanatorium treatment was not widely available to First Nations TB patients and was not pursued vigorously until the 1945 proposal, yet beds did not become available until 1950 [1]. Despite effective chemotherapy and the growing feasibility of outpatient treatment, institutionalization and isolation continued to be primary goals of First Nations healthcare policy in Saskatchewan [15]. Medical historians have hypothesized that such policies were devised to protect the interests of health care institutions and their employees as improvements in TB prevention and care made it difficult to justify ongoing allocation of resources to specialized hospitals and personnel [1]. Rising readmission rates (Fig. 6) confirm that new beds were indeed becoming available during the dual- and triple-therapy eras and also indicate that the antibiotic regimens were increasingly effective, with more patients surviving long enough to be readmitted. Consequently, the peak sanatoria admissions year for the entire Saskatchewan First Nations population occurred in 1953 [2]. Our analyses reveal a similar pattern, with both first and overall admissions peaking in 1954 at the three sanatoria in this dataset (Fig. 5). This coincides with increased availability of triple treatment and sanatorium beds made available to the First Nations population.

It is not surprising that we find overall admissions low and death rates high before the mid-1940s. It was known at the time that pre-chemotherapy therapies and monotherapy were not very effective [9, 16–18]. Dual therapy was slightly more effective, though the death rate was still higher than for triple therapy [13, 14, 19]. By the mid-1950s it was well established that triple therapy was effective, with INH being the most effective of the three antibiotics [13, 14, 20]. Medical historians have hypothesized that isolation in sanatoria was a key driver in decreasing TB incidence in this population [2]. We find some support for this hypothesis as death rates started decreasing in the mid-1940s, before more effective triple-therapy was available (Fig. 7). Medical histories [15, 21] and firsthand accounts describe the severe hardships associated with TB treatment for First Nations during this era [22, 23]. The difficulty of enduring such prolonged hospitalization is highlighted by our finding that as treatment length became regularized and admission lengths increased during the 1950s, the proportion of patients lost to follow up, categorized in this study as those who left against medical advice or were evicted, increased (Fig. 5). Additionally, it appears that most TB deaths occurred in the hospital, rather than at home, for patients originally hospitalized (Table 3, Fig. 5), highlighting the arduous and isolating nature of TB treatment in this era.

### Limitations

Our analyses reflect length of treatment, rather than length of illness, so our results reflect the effect of changing treatment methods on treatment length, rather than illness duration. Because the sanatorium records do not contain notes of specific treatment received, we have classified each treatment era by the year the drug was discovered. This may have induced classification error in individuals admitted near the cut-off years. We believe any classification error was small, since the average patient who completed therapy was admitted more than a year, well into the new treatment era and likely received at least some of the new antibiotic. The pooled regression model produced poor fit statistics, however it is unclear if these are an artifact of the pooling method or reflective of the model itself. We expect some poor fit due to nonlinearity of the data, and the associations from the model are still clear.

## Conclusion

We find that as TB treatment became more effective between 1933 and 1959, admissions to TB sanatoria increased and lengthened, readmission rates rose, and death rates fell in the study population. These results provide insight into how healthcare systems adapt to new treatment modalities and changing technologies. The healthcare system under study here adapted in a step-wise fashion, initially intensifying its use of existing resources (inpatient care) before shifting to the new treatment paradigm (outpatient care) enabled by novel antibiotic therapies. Health care systems must constantly adapt to new threats, such as the current COVID-19 pandemic, and to new opportunities afforded by advances in treatment and diagnostics. Historical analyses such as this one support the development of models of health care system adaptation, which can be used to understand changes as they occur and make to predictions for the future.

As noted above, medical historians have previously shown that health care policies applied in this era to First Nations and Métis peoples differed from those applied to white Canadians, with earlier provision of cutting-edge care to white patients with TB. Our study shows that First Nations patients with TB were subjected to prolonged hospitalizations at a time when white patients were shifting to outpatient treatment and contemporaneous recommendations advocated a shift away from institutionalization of TB patients. Falling death rates (Fig. 7) and a significant increase in successfully completed treatments (Table 3) demonstrate the improving efficacy of TB treatment regimens during this era. However, despite these population-level indicators of better public health outcomes, at the institution-level we find high rates of individuals discontinuing care (Table 3, Fig. 5). Our findings emphasize the role of health care systems in ramifying inequity, a phenomenon that is reflected in persistent disparities in health outcomes for populations targeted by discrimination [24–26]. TB has disproportionate impacts on underserved communities. Analyses such as this one can offer lessons in how health care systems with embedded inequities adapt to new technologies. As modern TB treatment continues to change and more effective antibiotic combinations are developed, it is important for health care systems to deliver the highest quality care to the communities that need it most.

TB is a complicated disease and population level interventions are especially difficult to prospectively study. Historical analyses, such as those presented in this paper, are essential for understanding the efficacy of past control efforts and to inform predictive models of future epidemics. For example, results from this study can be used to parameterize length of treatment, length of quarantine, and, in certain cases, duration of TB disease in full dynamical models of TB epidemics. The estimates presented here add to a rich TB literature derived from twentieth century data, a time of intense epidemiological study of TB.

## Supplementary Information

**Additional file 1: Figure S1.** Missingness patterns in predictor variables for regression model. **Table S1.** Rhat values for imputed variables that were missing data in original dataset. **Table S2.** Parameter estimates from pooled generalized linear regression model.

## Data Availability

The datasets supporting the conclusions of this article are available from the archives of the Saskatchewan Lung Association and the Bureau of Epidemiology registry files from the Library and Archives Canada, “Tuberculosis Morbidity and Mortality 1938-1963”, National Health and Welfare Volume 1224.

## Abbreviations

INH: Isonicotinic acid hydrazide aka isoniazid
PAS: Para-aminosalicylic acid
TB: Tuberculosis
TST: Tuberculin skin test
