# Supplementary material for: Tuberculosis sanatorium treatment at the advent of the chemotherapy era
Source: BMC Infect Dis. 2020 Nov 11;20:831. doi: 10.1186/s12879-020-05539-w (PMC7656493; doi:10.1186/s12879-020-05539-w)
Supplement: Supplementary file 1 — Additional file 1: Figure S1. Missingness patterns in predictor variables for regression model. Table S1. Rhat values for imputed variables that were missing data in original dataset. Table S2. Parameter estimates from pooled generalized linear regression model. [file 12879_2020_5539_MOESM1_ESM.docx]

**Supplement**

**Figure S1:** Missingness patterns in predictor variables for regression model.


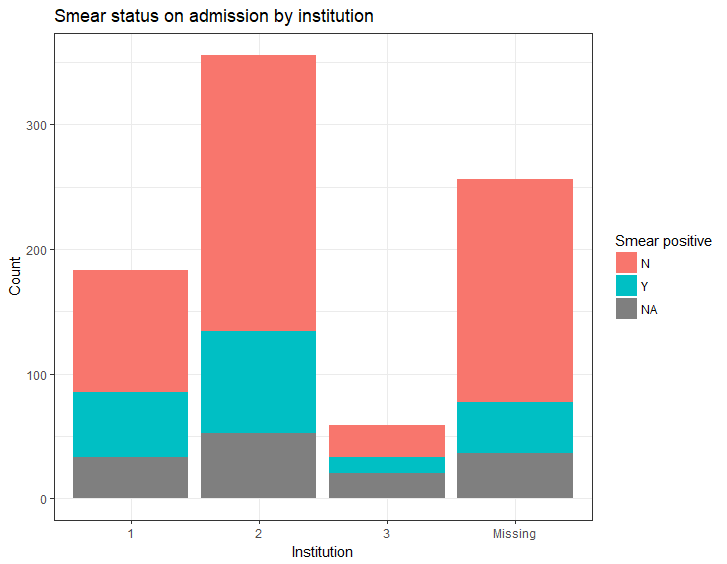

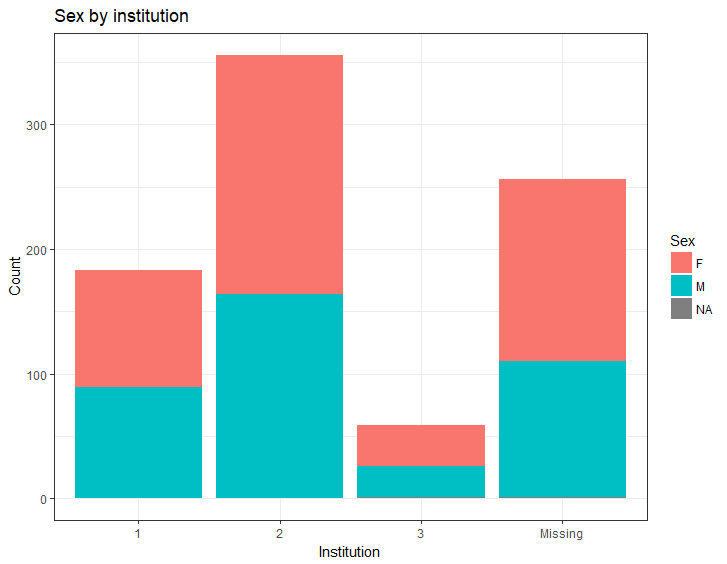

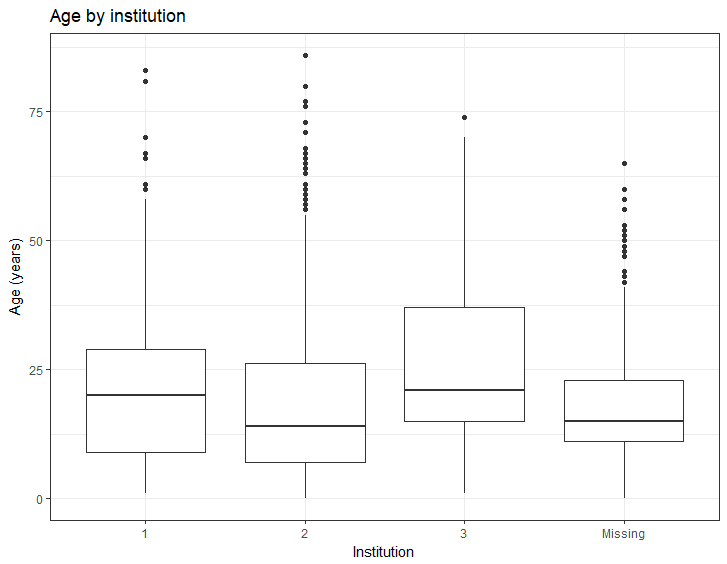

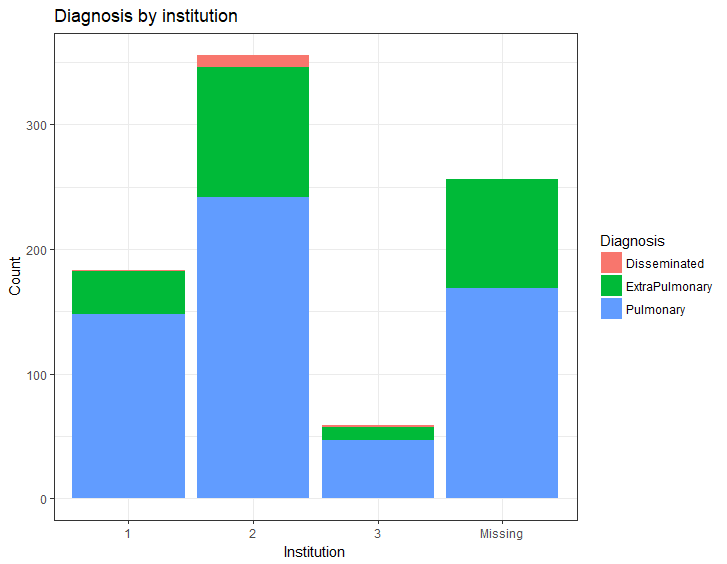

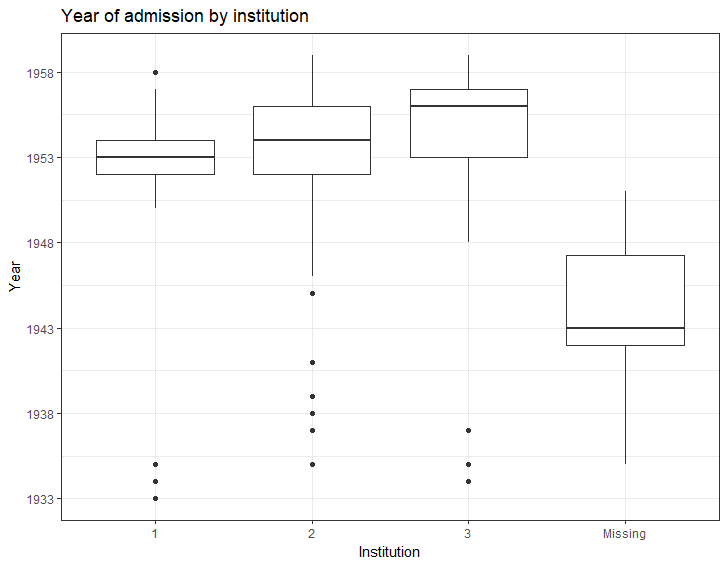

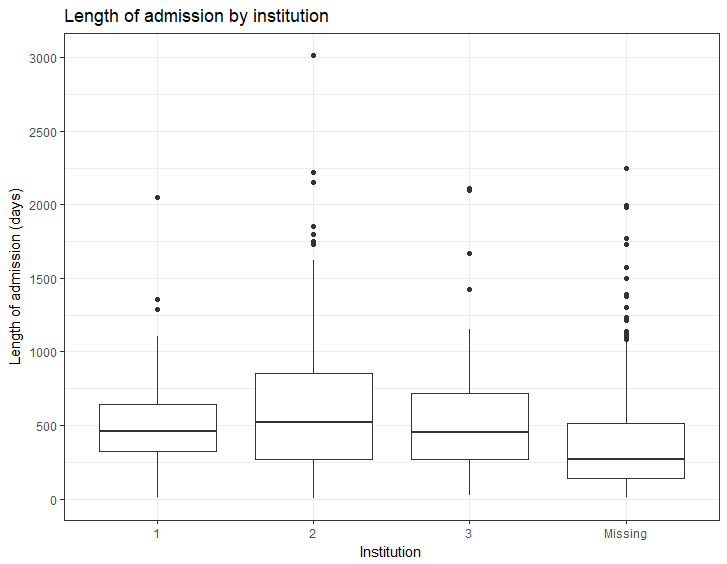


**Table S1:** Rhat values for imputed variables that were missing data in original dataset.

| Variable | Rhat for parameter means | Rhat for parameter standard deviations |
| --- | --- | --- |
| Institution | 0.998 | 0.998 |
| Age | 1.002 | 1.001 |
| Sex | 0.999 | 0.999 |
| Marital status | 1.003 | 1.003 |
| Open at admission | 1.001 | 1.000 |

**Table S2:** Parameter estimates from pooled generalized linear regression model.

| Variable | Estimate | SE | p-value |
| --- | --- | --- | --- |
| Intercept  (ref = Female, Pulmonary, Institution 2, smear negative) | 6.59 | 0.19 | 0.00 |
| Admission year (1933-1946) | 0.01 | 0.17 | 0.94 |
| Admission year (1946-1952) | -0.33 | 0.15 | 0.03 |
| Admission year (1952-1955) | -1.36 | 0.46 | 0.003 |
| Admission year (1955-1959) | -0.05 | 0.19 | 0.80 |
| Smear positive on admission | 0.35 | 0.06 | 0.00 |
| Smear positive on discharge | -0.21 | 0.20 | 0.31 |
| Age (centered at mean = 19.2 years) | -0.0006 | 0.002 | 0.78 |
| Sex (male) | -0.01 | 0.05 | 0.83 |
| Interaction age*sex (male) | -0.008 | 0.003 | 0.02 |
| Diagnosis (disseminated) | 0.12 | 0.19 | 0.52 |
| Diagnosis (extrapulmonary | 0.06 | 0.06 | 0.35 |
| Institution 1 | -0.23 | 0.07 | 0.002 |
| Institution 3 | -0.09 | 0.08 | 0.27 |

Note: outcome was log-transformed and the main predictor, year of admission, is nonlinear so variable estimates are not directly interpretable. The values above are useful in terms of directionality (positive = longer stay, negative = shorter stay) and relative to their standard error.
